# Thrombocytopenia and Bleeding in Patients With Oncologic Emergencies Following Cancer Therapy

**DOI:** 10.1002/cam4.71621

**Published:** 2026-02-10

**Authors:** Le Tian, Jia‐Xin Huang, Xi Zhang, Ning Li, Zhi‐Min Bian, Na Li, Shao‐Ming Wang, Xin‐Qi Liu, Zhi‐Yong Li, Qing‐Long Jiang, Chao Wang, Cong Zhao, Wei Wei, Ming‐Hua Cong

**Affiliations:** ^1^ Department of Comprehensive Oncology, National Cancer Center/National Clinical Research Center for Cancer/Cancer Hospital Chinese Academy of Medical Sciences and Peking Union Medical College Beijing China; ^2^ National Central Cancer Registry, National Cancer Center/National Clinical Research Center for Cancer/Cancer Hospital Chinese Academy of Medical Sciences and Peking Union Medical College Beijing China; ^3^ Department of Traditional Chinese Medicine National Cancer Center/National Clinical Research Center for Cancer/Cancer Hospital, Chinese Academy of Medical Sciences and Peking Union Medical College Beijing China

**Keywords:** bleeding, cancer, clinical outcome, emergency, thrombocytopenia

## Abstract

**Objective:**

There is a lack of studies investigating Cancer therapy‐induced thrombocytopenia (CTIT) and the risk factors predicting CTIT‐related hemorrhage in the emergency oncology patient population. This study aimed to present Chinese data on CTIT and organ bleeding in patients undergoing emergency oncology.

**Methods:**

This retrospective study was conducted in the Oncology Emergency Department. We evaluated the clinical features and outcomes of CTIT and associated organ hemorrhage.

**Results:**

A retrospective analysis collected data from 8590 cases of malignant tumor emergency visits. Among these, 1164 cases (13.5%) of CTIT met the inclusion criteria, with a median patient age of 61 years. 61 (5.24%) of the 1164 CTIT cases were associated with overt organ hemorrhage. Independent risk factors predicting CTIT deterioration included Eastern Cooperative Oncology Group (ECOG) score of 2–4 (odds ratio [OR] = 4.883), stage IV (OR = 2.275), organ bleeding (OR = 3.029), anemia (OR = 3.243), and fever (OR = 5.360), all with *p* < 0.05. Among the 61 cases with bleeding, 41% (25/61) involved lung cancer. The bleeding group had a significantly higher proportion of patients with fever (11.5% vs. 2.9%), pleural effusion (25.0% vs. 9.8%), and malnutrition requiring parenteral nutritional support (9.8% vs. 2.8%) compared to the non‐bleeding group (*p* < 0.05). Fever (OR = 4.886, *p* = 0.003) and pleural effusion (OR = 4.812, *p* = 0.007) were identified as independent risk factors for CTIT‐related bleeding.

**Conclusion:**

Malignancies associated with reduced platelet counts and additional risk factors require heightened clinical vigilance for hemorrhage development.

## 1 Introduction

Thrombocytopenia is a common complication of antineoplastic therapy [1], and thrombocytopenia‐induced hemorrhage remains one of the leading causes of mortality among oncology patients. This condition restricts the timely and optimal administration of antineoplastic drugs, increases the demand for transfusions, and exacerbates the medical and economic burden of care [2, 3, 4, 5, 6]. Large‐scale data on the clinical characteristics and treatment outcomes of emergency patients with Cancer therapy‐induced thrombocytopenia (CTIT) remain limited in China. Moreover, a reduced platelet count is not the sole factor contributing to organ bleeding in oncology patients, yet cohort studies specifically examining bleeding during CTIT are lacking. The clinical risk factors associated with organ bleeding in patients with CTIT have not been established. Therefore, this study aims to analyze the clinical characteristics of emergency CTIT patients, identify risk factors influencing their clinical outcomes, and assess both the characteristics and predictive factors of CTIT‐related bleeding.

## 2 Methods

A total of 8590 patients who visited the Emergency Department of Oncology at the Cancer Hospital of the Chinese Academy of Medical Sciences between January 2023 and May 2023 were retrospectively enrolled. Among them, 1164 patients met the inclusion criteria and were enrolled in the study. Follow‐up: The final follow‐up was conducted in May 2024.

The inclusion criteria: (1) A malignant tumor confirmed by pathological or cytological diagnosis at our hospital; (2) A Platelet count of < 100 × 10^9^/L following anti‐tumor therapy (including chemotherapy, radiotherapy, immunotherapy, or targeted therapy).

Exclusion criteria: (1) Absence of a confirmed malignant tumor diagnosis; (2) No history of antitumor treatment; (3) Thrombocytopenia due to other causes, such as aplastic anemia, acute leukemia, hypersplenism, and bone marrow metastases.

Treatment strategies included platelet‐raising drugs such as recombinant human thrombopoietin (rh‐TPO), interleukin‐11 (IL‐11), and TPO receptor analogs. Additional supportive measures comprised hemostatic agents, parenteral and intranasal nutritional support, anti‐infective therapy, hepatoprotective agents, and gastric acid suppression therapy. For patients with mild thrombocytopenia, clinical management will determine whether to observe or administer thrombopoietic agents based on the specific clinical condition.

Short‐term (28 days of follow‐up) clinical outcomes of disease regression were categorized as follows: (1) improvement: defined by the resolution or alleviation of symptoms, signs, and test marker abnormalities; (2) worsening of disease, characterized by worsening symptoms, signs, test markers, or death; and (3) hospital admission: necessitated by disease progression or other indications for hospitalization. The primary outcome was clinical worsening and the secondary outcome was hospital admission within 28 days of the emergency department visit.

### 2.1 Statistics

Statistical analyses were performed using IBM SPSS (Version 20.0. Armonk, NY: IBM Corp). Continuous variables were analyzed using the *T*‐test or Mann–Whitney U test, depending on their distribution. Categorical variables were reported as absolute numbers or percentages and compared using a χ^2^ test. Logistic regression analysis was performed to identify independent predictive risk factors. Statistical significance was set at a two‐tailed *p*‐value of < 0.05.

## 3 Results

### 3.1 Clinical Characteristics of CTIT Emergency Patients

A retrospective analysis was conducted on 8590 cases presented to the oncology emergency department at the Cancer Hospital of the Chinese Academy of Medical Sciences. The median patient age was 59 years, and 49.3% were female. Among these cases, 1164 (13.55%) developed thrombocytopenia as a complication of antitumor therapy, with a median age of 61 years (18–85) and a male‐to‐female ratio of 1.03:1. The median platelet count (Q1 Q3) across 1164 cases was 59 (39 77) × 10^9^/L. The median number (Q1 Q3) of platelets in the hemorrhage group and non‐hemorrhage groups was 48 × 10^9^/L (20 55) and 61 × 10^9^/L (40 78) respectively. Lung cancer was the most frequently observed tumor type in CTIT, accounting for 34.5% of cases. Adenocarcinoma was the most common pathological subtype (62.3%). Of the patients with CTIT, 96.6% had previously received chemotherapy, 8.3% had undergone treatment with immune checkpoint inhibitors, and 5.7% had received targeted therapy. Forty‐nine patients (4.2%) had undergone radiotherapy. Comorbid anemia was present in 16% (186) of CTIT cases, while leukopenia was observed in 58.8% (684). Overt organ bleeding occurred in 61 patients (5.24%), presenting as hemoptysis, hematemesis, hematochezia, hematuria, or vaginal bleeding. Platelet transfusions were administered to 2.5% (30/1164) of affected patients.

The clinical characteristics of the specific 1164 emergency patients with CTIT are shown in Table 1.

**TABLE 1:** The clinical characteristics of the specific 1164 emergency patients with CTIT and comparison of the clinical characteristics of the specific 61 patients with CTIT with organ hemorrhage.

Variable		Total (%)	Bleeding (%)	Non‐bleeding (%)	χ^2^	*p*
Sex	Male	593 (50.9)	31 (50.8)	562 (51)	0.000	0.984
	Female	571 (49.1)	30 (49.2)	541 (49)		
Year	≤ 65	797 (68.5)	46 (75.4)	751 (68.1)	1.436	0.231
	> 65	367 (31.5)	15 (24.6)	352 (31.9)		
ECOG	0–1	923 (79.3)	48 (78.7)	875 (79.3)	0.014	0.904
	2–4	241 (20.7)	13 (21.3)	228 (20.7)		
Tumor site					30.153	0.007
	Head and neck	16 (1.4)	0 (0)	16 (1.5)	0.897	1.000
	Esophagus	47 (4.1)	1 (1.6)	46 (4.2)	0.956	0.509
	Lung	399 (34.5)	25 (41.0)	374 (34.2)	1.285	0.257
	Thymus gland	11 (1)	0 (0)	11 (1.0)	0.614	1.000
	Gastric	86 (7.4)	5 (8.2)	81 (7.4)	0.061	0.800
	Colorectal	156 (13.5)	9 (14.8)	147 (13.4)	0.101	0.750
	Hepatobiliary	66 (5.7)	1 (1.6)	65 (5.9)	1.955	0.251
	Ovary	118 (10.2)	3 (4.9)	115 (10.5)	1.925	0.165
	Breast	67 (5.8)	1 (1.6)	66 (6.0)	2.011	0.253
	Cervix	48 (4.2)	2 (3.3)	46 (4.2)	0.116	1.000
	Lymphoma	46 (4.0)	0 (0)	46 (4.2)	2.649	0.168
	Kidney	2 (0.2)	0 (0)	2 (0.2)	0.111	1.000
	Bladder	1 (0.1)	0 (0)	1 (0.1)	0.055	1.000
	Pancreas	17 (1.5)	3 (4.9)	14 (1.3)	5.347	0.055
	Other or unknown primary	75 (6.5)	11 (18)	64 (5.9)	14.343	0.001
Pathological classification	Adenocarcinoma	530 (62.3)	37 (71.2)	493 (61.7)	4.932	0.424
	Squamous carcinoma	118 (13.9)	5 (9.6)	113 (14.1)		
	Small cell	87 (10.2)	7 (13.5)	80 (10.0)		
	Sarcoma	3 (0.4)	0 (0)	3 (0.4)		
	Lymphoma	25 (2.9)	0 (0)	25 (3.1)		
	Other	88 (10.3)	3 (5.8)	85 (10.6)		
Stage	I‐III	82 (7.0)	4 (6.6)	78 (7.1)	0.049	0.976
	IV	333 (28.6)	17 (27.9)	316 (28.6)		
Tumor control	CR/PR	2 (0.2)	0 (0)	2 (0.2)	0.246	0.884
	SD	610 (66.8)	35 (64.8)	575 (66.9)		
	PD	301 (33.0)	19 (35.2)	282 (32.8)		
Chemotherapy	Yes	1124 (96.6)	59 (96.7)	1065 (96.6)	0.005	0.945
	No	40 (3.4)	2 (3.3)	38 (3.4)		
Radiotherapy	Yes	49 (4.2)	2 (3.3)	47 (4.3)	0.138	0.710
	No	1115 (95.8)	59 (96.7)	1056 (95.7)		
Immunotherapy	Yes	97 (8.3)	5 (8.2)	92 (8.3)	0.002	0.968
	No	1067 (91.7)	56 (91.8)	1011 (91.7)		
Target therapy	Yes	66 (5.7)	6 (9.8)	60 (5.4)	2.089	0.148
	No	1098 (94.3)	55 (90.2)	1043 (94.6)		
Fever	Yes	39 (3.4)	7 (11.5)	32 (2.9)	13.123	< 0.001
	No	1125 (96.6)	54 (88.5)	1071 (97.1)		
Liver dysfunction	Yes	149 (12.8)	9 (14.8)	140 (12.7)	0.220	0.639
	No	1015 (87.2)	52 (85.2)	963 (87.7)		
Renal dysfunction	Cr > 97	35 (3.0)	2 (3.3)	33 (3.0)	0.016	0.898
	Cr ≤ 97	1129 (97)	59 (96.7)	1070 (97)		
Malignant plasmapheresis	Pleural effusion	32 (4.5)	5 (11.4)	27 (4.0)	9.954	0.007
	Ascite	45 (6.3)	6 (13.6)	39 (5.8)		
	No	635 (89.2)	33 (75)	602 (90.1)		
ALB (g/L)	≤ 35	108 (9.3)	8 (13.1)	100 (9.1)	1.126	0.289
	> 35	1056 (90.7)	53 (86.9)	1003 (90.9)		
Malnutrition requires PN	Yes	37 (3.2)	6 (9.8)	31 (2.8)	9.270	0.002
	No	1127 (96.8)	55 (90.2)	1072 (97.2)		
Anemia	Yes	186 (16.0)	12 (19.7)	174 (15.8)	0.654	0.419
	No	978 (84.0)	49 (80.3)	929 (84.2)		
Leucopenia	Yes	684 (58.8)	37 (60.7)	647 (58.7)	0.095	0.758
	No	480 (41.2)	24 (39.3)	456 (41.3)		
Raise PLT strategy	Rh‐TPO	852 (73.2)	45 (73.8)	807 (73.2)	40.292	< 0.001
	TPO‐RA	13 (1.1)	1 (1.6)	12 (1.1)		
	Rh‐TPO+ TPO‐RA	82 (7.0)	15 (24.6)	67 (6.1)		
	IL‐11	24 (2.1)	0 (0)	24 (2.2)		
	Observation	193 (16.6)	0 (0)	193 (17.5)		

Abbreviations: ALB, albumin; CR, complete regression; Cr, creatinine; CTIT, Cancer therapy‐induced thrombocytopenia; ECOG, Eastern Cooperative Oncology Group; IL‐11, interleukin‐11. Fever, underarm temperature ≥ 37.3°C. Liver dysfunction, ALT or AST levels exceeding the upper limit of normal reference values; PD, progressive disease; PLT, platelet; PN, parenteral nutrition; PR, partial response; Rh‐TPO, recombinant human thrombopoietin; SD, stable disease; TPO‐RA, thrombopoietin receptor agonists.

### 3.2 CTIT Clinical Treatment Regression

A total of 1164 CTIT cases were treated in the emergency department. Of these patients, 83.4% received thrombopoietic therapy, including 852 cases (73.1%) of rh‐TPO monotherapy and 82 cases (7.0%) of combined rh‐TPO and TPO‐RA therapy. Details are presented in Table 1. The clinical improvement rate among patients with CTIT was 96.7% (1126/1164), while 29 patients (2.5%) experienced disease progression. No short‐term mortality was observed. Following treatment with platelet‐stimulating medication, a total of 12 cases of thrombosis occurred, all involving lower limb venous thrombosis. Of these, 9 cases occurred in the rh‐TPO monotherapy group, 2 cases in the combination therapy group, and 1 case in the IL‐11 therapy group.

The rate of disease progression was significantly higher in patients with an ECOG score of 2–4 (7.1%) compared to those with an ECOG score of 0–1 (1.3%) (*p* < 0.001). Similarly, the deterioration rate was higher in patients with stage IV disease than in those with stages I–III (4.2% vs. 0%; *p* < 0.001). Patients with coexisting fever, anemia, and hypoproteinemia had a significantly higher rate of deterioration than those without these conditions (*p* < 0.05). Additionally, the proportion of patients showing clinical improvement was lower in those with concurrent renal insufficiency than in those without renal impairment (91.4% vs. 96.9%; *p* = 0.003). Furthermore, patients with concurrent organ hemorrhage had a higher deterioration rate (9.8%) compared to those without hemorrhage (2.1%; *p* = 0.001).

Specific CTIT emergency treatment regression characteristics are shown in Table 2.

**TABLE 2:** Specific CTIT emergency treatment regression characteristics.

Fact	Improvement (%)	Worse (%)	Hospitalization (%)	χ^2^	*p*
Total	1126 (96.7)	29 (2.5)	9 (0.8)		
Sex				5.08	0.079
Male	580 (97.8)	11 (1.9)	2 (0.3)		
Female	546 (95.6)	18 (3.2)	7 (1.2)		
Age				0.703	0.704
≤ 65	772 (96.9)	20 (2.5)	5 (0.6)		
> 65	354 (96.5)	9 (2.5)	4 (1.1)		
ECOG				33.133	< 0.001
0–1	907 (98.3)	12 (1.3)	4 (0.4)		
2–4	219 (90.9)	17 (7.1)	5 (2.1)		
Stage				22.065	< 0.001
I‐III	79 (96.3)	0 (0)	3 (3.7)		
IV	314 (94.3)	14 (4.2)	5 (1.5)		
unknown	733 (97.9)	15 (2.0)	1 (0.1)		
Tumor control				3.239	0.519
CR/PR	2 (100)	0 (0)	0 (0)		
SD	589 (96.6)	15 (2.5)	6 (1.0)		
PD	284 (94.4)	14 (4.7)	3 (1.0)		
Bleeding				14.723	0.001
Yes	55 (90.2)	6 (9.8)	0 (0)		
No	1071 (97.1)	23 (2.1)	9 (0.8)		
Leucopenia				3.018	0.221
Yes	659 (96.3)	21 (3.1)	4 (0.6)		
No	467 (97.3)	8 (1.7)	5 (1.0)		
Anemia				18.764	< 0.01
Yes	171 (91.9)	13 (7.0)	2 (1.1)		
No	955 (97.6)	16 (1.6)	7 (0.7)		
Liver dysfunction				2.948	0.229
Yes	143 (96.0)	6 (4.0)	0 (0)		
No	983 (96.8)	23 (2.3)	9 (0.9)		
Renal dysfunction				11.519	0.003
Yes	32 (91.4)	1 (2.9)	2 (5.7)		
No	1094 (96.9)	28 (2.5)	7 (0.6)		
Fever				29.497	< 0.001
Yes	32 (82.1)	6 (15.4)	1 (2.6)		
No	1094 (97.2)	23 (2.0)	8 (0.7)		
ALB (g/L)				13.781	0.001
≤ 35	98 (90.7)	8 (7.4)	2 (1.9)		
> 35	1028 (97.3)	21 (2.0)	7 (0.7)		

Abbreviations: ALB, albumin; CR, complete regression; CTIT, Cancer therapy‐induced thrombocytopenia; ECOG, Eastern Cooperative Oncology Group; PD, progressive disease; PR, partial response; SD, stable disease.

### 3.3 Risk Factors Affecting CTIT Regression

Combined organ hemorrhage independently influenced clinical outcomes in patients with CTIT (odds ratio [OR] = 3.029, 95% confidence interval [CI]: 1.049–8.749, *p* = 0.041). Additionally, ECOG score of 2–4, stage IV disease, anemia, and fever were independent risk factors for treatment deterioration in emergency CTIT patients.

**FIGURE 1:**
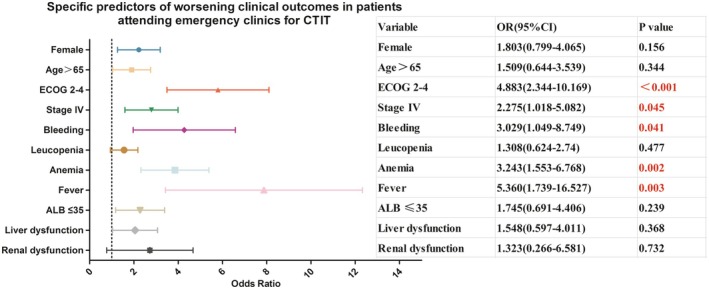
Specific predictors of worsening clinical outcomes in patients attending emergency clinics for CTIT.

Specific predictors of worsening clinical outcomes in patients attending emergency clinics for CTIT are presented in Table 3 and Figure 1.

**TABLE 3:** Factors predicting worsening clinical outcomes in patients attending an emergency department for cancer therapy induced thrombocytopenia.

Factor	Multivariate	
	OR (95% CI)	*P*
Sex		
Male	Reference	
Female	1.803 (0.799–4.065)	0.156
Age		
≤ 65	Reference	
> 65	1.509 (0.644–3.539)	0.344
ECOG		
0–1	Reference	
2–4	4.883 (2.344–10.169)	< 0.001
Stage		
I‐III	Reference	
IV	2.275 (1.018–5.082)	0.045
unknown	2.236 (0.596–8.395)	0.233
Bleeding		
Yes	3.029 (1.049–8.749)	0.041
No	Reference	
Leucopenia		
Yes	1.308 (0.624–2.74)	0.477
No	Reference	
Anemia		
Yes	3.243 (1.553–6.768)	0.002
No	Reference	
Fever		
Yes	5.360 (1.739–16.527)	0.003
No	Reference	
ALB (g/L)		
≤ 35	1.745 (0.691–4.406)	0.239
> 35	Reference	
Liver dysfunction		
Yes	1.548 (0.597–4.011)	0.368
No	Reference	
Renal dysfunction		
Yes	1.323 (0.266–6.581)	0.732
No	Reference	

Abbreviations: ECOG, Eastern Cooperative Oncology Group; ALB, albumin; Fever, underarm temperature ≥ 37.3°C; Liver dysfunction, ALT or AST levels exceeding the upper limit of normal reference values; Renal dysfunction, Cr > 97 umol/L.

### 3.4 Clinical Features of Cases of CTIT Combined With Visceral Overt Hemorrhage

Definition of visceral overt hemorrhage: Clinically observable bleeding symptoms include hemoptysis, hematemesis, hematochezia, hematuria, and vaginal bleeding. Skin hemorrhages and asymptomatic cases with occult blood positivity were excluded.

Among 1164 cases of CTIT, 61 (5.24%) were associated with overt visceral bleeding. Sixteen of these patients required red blood cell transfusions. The most common tumor site among patients with CTIT‐related hemorrhage was the lung (41%, 25/61). The incidence of concurrent fever was significantly higher in the hemorrhage group than in the non‐hemorrhagic group (11.5% vs. 2.9%, *p* < 0.001). Similarly, pleural and abdominal effusions were more prevalent in the hemorrhage group than in the non‐hemorrhage group (11.4% vs. 4.0% and 13.6% vs. 5.8%, respectively; *p* = 0.007). Additionally, a higher proportion of patients in the hemorrhage group required parenteral nutritional support due to malnutrition compared to the non‐hemorrhage group (9.8% vs. 2.8%; *p* = 0.002). The overall improvement rates were 90.2% (55/61) in the hemorrhage group and 97.1% (1071/1103) in the non‐hemorrhage group (*p* = 0.001).

There was no statistically significant difference between the CTIT bleeding and non‐bleeding groups in terms of tumor stage, antitumor efficacy, hepatic and renal insufficiency, anemia, or leukopenia.

A comparison of the clinical characteristics of the specific 61 patients with CTIT with organ hemorrhage is shown in Table 1.

### 3.5 Risk Factors Predicting Organ Hemorrhage in CTIT

An ovarian tumor was identified as an independent protective factor against organ hemorrhage in patients with CTIT (odds ratio [OR] = 0.081, 95% confidence interval [CI]: 0.009–0.696, *p* = 0.022). Febrile symptoms were found to be an independent risk factor for organ hemorrhage in CTIT (odds ratio [OR] = 4.886, 95% confidence interval [CI]: 1.692–14.114, *p* = 0.003). Pleural effusion was determined to be an independent risk factor for the development of organ hemorrhage on CTIT (odds ratio [OR] = 4.812, 95% confidence interval [CI]: 1.546–14.975, *p* = 0.007).

**FIGURE 2:**
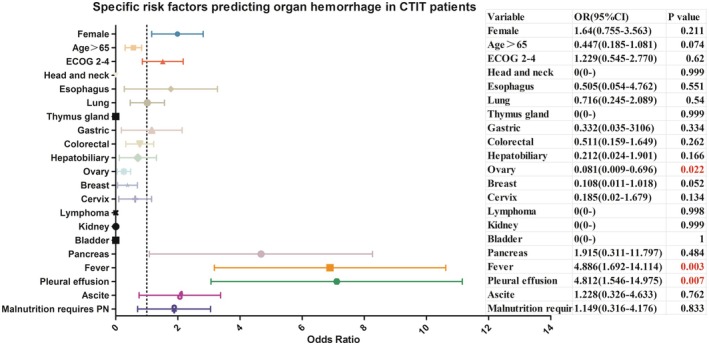
Specific risk factors predicting organ hemorrhage in CTIT patients.

Specific risk factors predicting organ hemorrhage in CTIT patients are presented in Table 4 and Figure 2.

**TABLE 4:** Risk factors for predicting organ hemorrhage in patients with cancer therapy induced thrombocytopenia.

Factor		Univariate		Multivariate	
		OR (95% CI)	*p*	OR (95% CI)	*p*
Gender	Male	Reference		Reference	
	Female	1.005 (0.6–1.684)	0.984	1.64 (0.755–3.563)	0.211
Age	≤ 65	Reference		Reference	
	> 65	0.696 (0.383–1.263)	0.233	0.447 (0.185–1.081)	0.074
ECOG	0–1	Reference		Reference	
	2–4	1.039 (0.554–1.951)	0.904	1.229 (0.545–2.770)	0.620
Tumor site	Head and neck	—	0.998	—	0.999
	Esophagus	0.126 (0.016–1.014)	0.052	0.505 (0.054–4.762)	0.551
	Lung	0.389 (0.182–0.829)	0.014	0.716 (0.245–2.089)	0.54
	Thymus gland	—	0.999	—	0.999
	Gastric	0.359 (0.119–1.086)	0.07	0.332 (0.035–3.106)	0.334
	Colorectal	0.356 (0.141–0.902)	0.029	0.511 (0.159–1.649)	0.262
	Hepatobiliary	0.09 (0.011–0.714)	0.023	0.212 (0.024–1.901)	0.166
	Ovary	0.152 (0.041–0.564)	0.005	0.081 (0.009–0.696)	0.022
	Breast	0.088 (0.011–0.703)	0.022	0.108 (0.011–1.018)	0.052
	Cervix	0.253 (0.054–1.196)	0.083	0.185 (0.02–1.679)	0.134
	Lymphoma	—	0.997	—	0.998
	Kidney	—	0.999	—	0.999
	Bladder	—	1.0	—	1.0
	Pancreas	1.247 (0.307–5.063)	0.758	1.915 (0.311–11.797)	0.484
	Other or unknown primary	Reference		Reference	
Stage	I‐III	Reference			
	IV	0.909 (0.317–2.608)	0.859		
	unknown	0.954 (0.532–1.708)	0.873		
Tumor control	CR/PR	Reference			
	SD	—	0.999		
	PD	—	0.999		
Fever	Yes	4.339 (1.832–10.276)	0.001	4.886 (1.692–14.114)	0.003
	No	Reference		Reference	
ALB (g/L)	≤ 35	1.514 (0.7–3.274)	0.292		
	> 35	Reference			
Liver dysfunction	Yes	1.19 (0.574–2.469)	0.639		
	No	Reference			
Renal dysfunction	Yes	1.099 (0.258–4.691)			
	No	Reference			
Malignant plasmapheresis	Pleural effusion	3.378 (1.222–9.336)	0.019	4.812 (1.546–14.975)	0.007
	Ascite	2.807 (1.109–7.1)	0.029	1.228 (0.326–4.633)	0.762
	No	Reference		Reference	
Malnutrition requires parenteral nutrition	Yes	3.772 (1.511–9.421)	0.004	1.149 (0.316–4.176)	0.833
	No	Reference		Reference	

Abbreviations: ALB, albumin; CR, complete regression; CTIT, Cancer therapy‐induced thrombocytopenia; ECOG, Eastern Cooperative Oncology Group; Fever, underarm temperature ≥ 37.3°C; Liver dysfunction, ALT or AST levels exceeding the upper limit of normal reference values. Renal dysfunction, Cr > 97 umol/L; PD, progressive disease; PR, partial response; SD, stable disease.

## 4 Discussion

Lack scale cohort data on oncological emergency cases treated with CTIT remain limited. In a retrospective analysis of patients with solid tumors receiving first‐ or second‐line treatment, the incidence of chemotherapy‐induced severe thrombocytopenia (CIT) (platelet count < 75 G/L) was 10.1%, with lung and bladder cancers exhibiting the highest rates of CIT [7]. In this study, nearly one in seven oncology emergency cases involved antitumor therapy‐related thrombocytopenia, with lung cancer being the most frequently associated malignancy, consistent with previous findings. This may be attributed to the high prevalence of lung cancer in China and the common use of chemotherapy regimens containing carboplatin, gemcitabine, and other thrombocytopenia‐inducing agents [8]. Due to the variety of tumor types and the complexity of antitumor regimens in the emergency cases analyzed, specific chemotherapy drugs were not included in the assessment of CTIT cases. Future research will focus on analyzing single‐tumor types and incorporating additional antitumor factors, such as specific chemotherapy regimens.

The incidence of CTIT‐related organ bleeding in this study was 5.24%, while the platelet transfusion rate was 2.5%. A retrospective cross‐sectional study evaluating the efficacy of various platelet‐raising therapies for CTIT [4] reported that among 1437 patients, 49.3% received rhTPO alone, with a platelet transfusion rate of 8.1%. In contrast, this study included all emergency visits for CTIT demonstrated a higher rate of improvement following pharmacological treatment and a lower platelet transfusion rate than the previous study. Another retrospective study investigating CTIT used compared the efficacy and cost‐effectiveness of rhTPO and interleukin‐11, concluding that while both treatments had similar efficacy, interleukin‐11 was more cost‐effective [6]. Additionally, a study of 112 patients with immune thrombocytopenia (ITP) with severe bleeding [9] reported a mortality rate of 30.6% and identified a lack of consensus regarding the prognostic impact of individualized treatments. That the study highlighted the need for a standardized approach to managing ITP with severe bleeding. Notably, no patients in this study died from CTIT‐related bleeding, and long‐term follow‐up will be conducted to clarify differences in long‐term prognosis. A multicenter prospective cohort study on thrombocytopenia in patients in the intensive care unit (ICU) [10] reported that 23.4% of patients had thrombocytopenia at ICU admission and identified hemorrhage as risk a factor for its progression. Similarly, the present study analyzed oncologic emergency oncology cases of CTIT and found that concurrent bleeding was a risk factor for disease. These findings indicate that in critically ill patients, thrombocytopenia accompanied by bleeding is a sign of a severe condition requiring proactive prevention and clinical management.

This study identified combined organ bleeding as an independent risk factor influencing the short‐term outcomes of patients undergoing CTIT. A previous study examining bleeding due to CIT in solid tumors [5] found that bleeding was associated with worse overall survival (OS). The study also concluded that although the overall incidence of bleeding in CIT was low, it exceeded 20% in specific subgroups with relevant clinical characteristics. However, the study did not establish clinical prediction rules for CIT‐related hemorrhages. In our study, the incidence of organ hemorrhage in CTIT was 5.24%. Fever was identified as an independent risk factor for both the clinical outcomes of CTIT and the prediction of organ hemorrhage. Among the patients in our study, 58.8% of CTIT cases were accompanied by leukopenia. Additionally, 94.9% (35/39) of CTIT cases with fever were diagnosed with microbial infections. It is possible that these infections exacerbated bone marrow suppression, accelerated platelet clearance, and shortened platelet lifespan [11, 12], thereby increasing the risk of hemorrhage. A separate study [13] identified age > 65 years, hepatic or renal insufficiency, malnutrition, and co‐infections as risk factors for severe and persistent chemotherapy‐induced myelosuppression. The findings of our study align with those results, as multiple studies have demonstrated that malnutrition exacerbates myelosuppressive toxicity related to antitumor therapy [14, 15]. Additionally, our study found that patients with renal insufficiency had a lower CTIT improvement rate than those with normal renal function. Renal insufficiency negatively affected short‐term CTIT outcomes, and the incidence of bleeding was higher in malnourished patients with CTIT who required parenteral nutritional support. However, neither renal insufficiency nor malnutrition was identified as an independent predictor of CTIT outcomes or bleeding. A possible explanation is that concurrent renal and hepatic impairment affects the clearance and metabolism of chemotherapeutic agents, prolonging their presence in the body and increasing myelosuppressive toxicity [13].

We identified clinical risk factors associated with bleeding in CTIT; however, no definitive drugs have been established for its prevention. A UK‐based, randomized, double‐blind, phase 3 trial [16] comparing tranexamic acid to placebo for the prevention of severe thrombocytopenic bleeding in hematological malignancies found insufficient evidence to support that the routine use of tranexamic acid in reducing bleeding incidence in those receiving high‐dose chemotherapy. In China, Yunnan Baiyao is empirically recommended for hemostasis in patients with organ hemorrhage. Yunnan Baiyao is a traditional Chinese medicine [17, 18], and its potential role in preventing CTIT‐related hemorrhage warrants further investigation.

This study had some limitations. First, certain medicines promoting platelet production in this study are only available within the Chinese context (rhTPO and IL‐11), thereby limiting the possibility of extrapolating the findings to other settings. Secondly, this study did not classify or analyze the antitumor regimens, specific drugs, or the number of treatment cycles administered prior to CTIT. This omission was due to the complexity and diversity of tumor sites and pathological types in emergency cancer patients, as well as the wide variety of treatment regimens available. Consequently, an analysis of specific therapeutic drugs was not conducted at this stage. However, future research will focus on evaluating different treatment regimens for specific tumor types to assess their impact on bleeding in CTIT and to determine the effect of delayed antitumor therapy. Additionally, as a retrospective cohort study, this research primarily examines the current status and recent outcomes of CTIT and bleeding in Chinese patients with oncological emergencies. However, long‐term survival data for all patients have not yet been collected; future investigations will further explore the impact of CTIT and bleeding on the long‐term prognosis of patients with different tumor types.

Finally, emergency oncology is an emerging interdisciplinary field in China, and our center is the first in the country to establish an emergency oncology department. Additionally, it has been designated as a National Clinical Key Specialty in Emergencies. Although this study is a single‐center retrospective cohort study, emergency oncology cohort data in China remain scarce. Therefore, this study analyzed the emergency oncology data of CTIT to contribute to advancing the oncology emergency medicine field and to guide future prospective cohort studies on emergency CTIT.

## 5 Conclusion

CTIT cases constitute approximately one in seven oncologic emergencies in China. Poor physical condition, fever, organ hemorrhage, and anemia are identified as risk factors for short‐term CTIT regression. Malignancy cases with thrombocytopenia and additional risk factors necessitate heightened clinical vigilance for hemorrhage development.

## Author Contributions

**Le Tian:** conceptualization (equal), funding acquisition (equal), methodology (equal), resources (equal), software (lead), visualization (lead), writing – original draft (lead), writing – review and editing (lead). **Jia‐Xin Huang:** formal analysis (equal), validation (equal). **Xi Zhang:** data curation (equal), supervision (supporting). **Ning Li:** investigation (equal), resources (supporting). **Zhi‐Min Bian:** software (supporting). **Na Li:** project administration (supporting). **Shao‐Ming Wang:** funding acquisition (supporting). **Xin‐Qi Liu:** supervision (supporting). **Zhi‐Yong Li:** data curation (supporting), resources (supporting). **Qing‐Long Jiang:** conceptualization (supporting), investigation (equal), methodology (equal), project administration (equal). **Chao Wang:** resources (equal), software (equal). **Cong Zhao:** software (supporting), supervision (supporting). **Wei Wei:** methodology (supporting), resources (supporting). **Ming‐Hua Cong:** conceptualization (equal), data curation (supporting), supervision (equal), writing – review and editing (lead).

## Funding

This study was supported by the National High Level Hospital Clinical Research Funding (80102022524).

## Ethics Statement

This study was in accordance with the principles of the Declaration of Helsinki and was approved by the Medical Ethics Committee of the Cancer Hospital, Chinese Academy of Medical Science (reference number: 24–208/4488). All participants were informed of the purpose of the study and provided signed informed consent. All methods were performed in accordance with the relevant guidelines and regulations.

## Consent

The authors have nothing to report.

## Conflicts of Interest

The authors declare no conflicts of interest.

## Data Availability

All data generated or analyzed during this study are included in this published article.

## References

[1] J. Mei , F. Jiao , Y. Li , J. Cui , H. Yang , and L. Wang , “Application of Thrombopoietic Agents in Cancer Therapy‐Induced Thrombocytopenia: A Comprehensive Review,” Blood Reviews 70 (2025): 101257, 10.1016/j.blre.2025.101257. 39809679

[2] N. Denduluri , G. H. Lyman , Y. Wang , et al., “Chemotherapy Dose Intensity and Overall Survival Among Patients With Advanced Breast or Ovarian Cancer,” Clinical Breast Cancer 18, no. 5 (2018): 380–386, 10.1016/j.clbc.2018.02.003. 29622384

[3] A. Gao , L. Zhang , and D. Zhong , “Chemotherapy‐Induced Thrombocytopenia: Literature Review,” Discover Oncology 14, no. 1 (2023): 10, 10.1007/s12672-023-00616-3. 36695938 PMC9877263

[4] M. Chen , L. Li , Q. Xia , et al., “A Real‐World Observation on Thrombopoietic Agents for Patients With Cancer Treatment‐Induced Thrombocytopenia in China: A Multicenter, Cross‐Sectional Study,” Cancer 130, no. S8 (2024): 1524–1538, 10.1002/cncr.35292. 38515388

[5] L. S. Elting , E. B. Rubenstein , C. G. Martin , et al., “Incidence, Cost, and Outcomes of Bleeding and Chemotherapy Dose Modification Among Solid Tumor Patients With Chemotherapy‐Induced Thrombocytopenia,” Journal of Clinical Oncology 19, no. 4 (2001): 1137–1146, 10.1200/JCO.2001.19.4.1137. 11181679

[6] F. M. Gong , F. Y. Liu , X. Ma , et al., “Effectiveness and Economic Evaluation of rhTPO and rhIL‐11 in the Treatment of Cancer Therapy Induced Thrombocytopenia Based on Real‐World Research,” Frontiers in Pharmacology 15 (2024): 1288964, 10.3389/fphar.2024.1288964. 38327986 PMC10848320

[7] A. Hitron , D. Steinke , S. Sutphin , A. Lawson , J. Talbert , and V. Adams , “Incidence and Risk Factors of Clinically Significant Chemotherapy‐Induced Thrombocytopenia in Patients With Solid Tumors,” Journal of Oncology Pharmacy Practice 17, no. 4 (2011): 312–319, 10.1177/1078155210380293. 20823048

[8] F. Griesinger , E. E. Korol , S. Kayaniyil , N. Varol , T. Ebner , and S. M. Goring , “Efficacy and Safety of First‐Line Carboplatin‐Versus Cisplatin‐Based Chemotherapy for Non‐Small Cell Lung Cancer: A Meta‐Analysis,” Lung Cancer 135 (2019): 196–204, 10.1016/j.lungcan.2019.07.010. 31446995

[9] S. R. Chowdhury , E. Sirotich , G. Guyatt , et al., “Treatment of Critical Bleeds in Patients With Immune Thrombocytopenia: A Systematic Review,” European Journal of Haematology 114, no. 3 (2025): 458–468, 10.1111/ejh.14351. 39552264 PMC11798764

[10] C. T. Anthon , F. Pène , A. Perner , et al., “Thrombocytopenia and Platelet Transfusions in ICU Patients: An International Inception Cohort Study (PLOT‐ICU),” Intensive Care Medicine 49, no. 11 (2023): 1327–1338, 10.1007/s00134-023-07225-2. 37812225 PMC10622358

[11] Chinese Society of Clinical Oncology Guidelines Working Committee , CSCO Guidelines for the Diagnosis and Treatment of Thrombocytopenia Caused by Oncological Treatment 2024 (People's Health Publishing House, 2023).

[12] D. J. Kuter and G. S. Tillotson , “Hematologic Effects of Antimicrobials: Focus on the Oxazolidinone Linezolid,” Pharmacotherapy 21, no. 8 (2001): 1010–1013, 10.1592/phco.21.11.1010.34517. 11718489

[13] R. Alyamany , A. Alnughmush , H. Alzahrani , and M. Alfayez , “Let It Grow: The Role of Growth Factors in Managing Chemotherapy‐Induced Cytopenia,” Current Oncology 31, no. 12 (2024): 8094–8109, 10.3390/curroncol31120596. 39727719 PMC11675056

[14] A. Pérez‐Pitarch , B. Guglieri‐López , A. Nacher , V. Merino , and M. Merino‐Sanjuán , “Impact of Undernutrition on the Pharmacokinetics and Pharmacodynamics of Anticancer Drugs: A Literature Review,” Nutrition and Cancer 69, no. 4 (2017): 555–563, 10.1080/01635581.2017.1299878. 28353359

[15] Q. Zhou , H. Yang , X. Wang , et al., “The Dynamic Effects of Nutritional Status on Chemotherapy‐Related Toxicity in Patients With Non‐Hodgkin's Lymphoma,” European Journal of Clinical Nutrition 79 (2025): 452–459, 10.1038/s41430-025-01565-6. 39810008

[16] TREATT Trial Investigators , “Tranexamic Acid Versus Placebo to Prevent Bleeding in Patients With Haematological Malignancies and Severe Thrombocytopenia (TREATT): A Randomised, Double‐Blind, Parallel, Phase 3 Superiority Trial,” Lancet Haematology 12, no. 1 (2025): e14–e22, 10.1016/S2352-3026(24)00317-X. 39642900

[17] N. Zhang , K. Guo , W. Lin , et al., “Yunnan Baiyao Exerts Anti‐Glioma Activity by Inducing Autophagy‐Dependent Necroptosis,” Journal of Ethnopharmacology 335 (2024): 118658, 10.1016/j.jep.2024.118658. 39103023

[18] E. J. Ladas , J. B. Karlik , D. Rooney , et al., “Topical Yunnan Baiyao Administration as an Adjunctive Therapy for Bleeding Complications in Adolescents With Advanced Cancer,” Supportive Care in Cancer 20, no. 12 (2012): 3379–3383, 10.1007/s00520-012-1598-1. 23052909

